# Effect of bacteriophages on growth performance and health indicators in broiler chickens in the absence of bacterial challenge – A review

**DOI:** 10.17221/5/2025-VETMED

**Published:** 2026-02-27

**Authors:** Mohd Asrore Mohd Shaufi, Suet Lin Chia, Hasliza Abu Hassim, Mohd Termizi Yusof, Muhammad Afiq Akbar, Adelene Ai-Lian Song, Shuhaimi Mustafa

**Affiliations:** ^1^Department of Microbiology, Faculty of Biotechnology and Biomolecular Sciences, University of Putra Malaysia, Serdang, Selangor, Malaysia; ^2^Research Laboratory of Probiotics and Cancer Therapeutics, UPM-MAKNA Cancer Research Laboratory (CANRES), Institute of Bioscience, University of Putra Malaysia, Serdang, Selangor, Malaysia; ^3^Institute of Tropical Agriculture and Food Security, University of Putra Malaysia, Serdang, Selangor, Malaysia

**Keywords:** antibiotic alternatives, feed efficiency, gut microbiota, phage formulation, poultry health

## Abstract

This review examines bacteriophages as alternatives to antibiotic growth promoters (AGPs) in broiler chickens, with a unique focus on effects observed in studies without experimental bacterial challenges. Driven by global antimicrobial resistance (AMR) concerns and sustainable poultry production demands, phage supplementation, a targeted strategy, potentially improves growth performance and gut health by preserving beneficial microbiota. This literature analysis assesses phage efficacy in healthy broilers under non-challenge conditions, evaluating key variables such as phage concentration, delivery, and targets, as well as outcomes such as feed conversion ratio (FCR) and gut health markers. Crucially, this review extends beyond efficacy to explore phage immunomodulatory capabilities, outlines optimisation strategies, and addresses risks and mitigation. Key findings show mixed efficacy of phages in non-challenged broilers: significant improvements in FCR and gut health were generally observed with high phage concentrations (e.g., ≥10^8^ PFU/g) and continuous delivery protocols, whereas lower doses yielded inconsistent or minimal benefits. Furthermore, choosing which bacteria phages target – for example, aiming at pathogens like *Salmonella* or managing common gut bacteria such as *E. coli –* greatly impacts outcomes. While phages show considerable potential as AGP alternatives, the review highlights that further research is vital to resolve inconsistencies, standardise protocols, and understand host genetic and environmental influences to optimise their commercial application.

## INTRODUCTION

Poultry meat is the most widely consumed type of meat globally. In 2021, its consumption reached an unprecedented 132.3 million tonnes, surpassing that of all other meat types, including pork, reflecting a growing global appetite for poultry products (Shahbandeh 2024). To meet this increasing demand, the poultry industry has rapidly evolved. Modern poultry production often resembles a factory-like setting, where chickens are raised in dense, sometimes unsanitary conditions. Unfortunately, these environments can serve as breeding grounds for disease, negatively impacting the health and growth of chickens.

Historically, since the 1940s, the poultry industry has depended heavily on the use of antibiotic growth promoters (AGPs). Administered in subtherapeutic amounts, AGPs were initially used as a prophylactic measure against diseases in chickens, and their introduction marked a significant boost in growth performance and overall farm productivity (Dibner and Richards 2005). However, this widespread practice has come under scrutiny for its role in the emergence and spread of antimicrobial-resistant (AMR) bacteria, posing a substantial threat to public health.

In response to rising concerns about AMR, there has been a global movement to eliminate AGP use in poultry farming. This shift includes legislative actions in various regions, with Sweden implementing a ban as early as 1986, followed by the European Union in 2006, South Korea in 2011, Australia in 2013, Thailand in 2015, China in 2016, Indonesia in 2018, and India in 2019 (Cuong et al. 2021; Abreu et al. 2023; Hibbard et al. 2023).

However, the phasing out of antibiotic growth promoters (AGPs) has introduced significant challenges for the poultry industry. Economic strains, including reduced growth rates and heightened feed conversion ratios, have coincided with a resurgence of enteric diseases such as necrotic enteritis (primarily caused by *Clostridium perfringens*) and coccidiosis (linked to *Eimeria* spp.) in commercial flocks (Adhikari et al. 2020; Emami and Dalloul 2021). Furthermore, AGP withdrawal has been associated with increased colonisation of zoonotic pathogens that threaten human health. For instance, in Sichuan Province, China, post-AGP ban surveillance revealed a rise in *Campylobacter jejuni* colonisation in swine, increasing from 41.3% pre-ban (2018–2019) to 46.8% post-ban (2020–2021) (Wen et al. 2022). Similarly, in broilers, AGP-free diets have been linked to higher *Salmonella enterica* shedding than AGP-supplemented diets. For example, Iqbal et al. (2023) demonstrated that avilamycin (an AGP) significantly reduced *Salmonella* Enteritidis shedding in faecal samples compared to control and probiotic-fed groups, with the AGP group showing the lowest bacterial counts in later trial stages (weeks 5–6), despite a transient parity with probiotics in week 4. These findings demonstrate AGPs’ historical role in suppressing pathogens, but also highlight the unintended consequences of their removal. Consequently, identifying safe and effective AGP alternatives is critical to simultaneously mitigate AMR and enteric disease risks.

The gastrointestinal tract (GIT) of poultry harbours a vast and diverse community of microorganisms, collectively known as the gut microbiota, which includes bacteria, archaea, fungi, and viruses (Yue et al. 2024). This complex ecosystem is fundamental to the host’s health, influencing nutrient digestion and absorption, immune system development and function, and resistance against pathogen colonisation (Khan et al. 2020). The composition of the gut microbiota is dynamic, changing significantly with the bird’s age, genetic background, diet, and rearing environment (Naeem and Bourassa 2025). Major bacterial phyla dominating the chicken gut include Firmicutes, Bacteroidetes, Proteobacteria, and Actinobacteria (Naeem and Bourassa 2025). This microbial community contains both beneficial and potentially harmful bacteria, summarised in Table 1. Maintaining a healthy balance within this complex gut microbiota is therefore essential for poultry health and productivity (Naeem and Bourassa 2025). Disruptions that allow pathogenic bacteria such as *Salmonella*, *Campylobacter*, Avian pathogenic *E. coli* (APEC), or *C. perfringens* to proliferate can lead to disease and increase the risk of transmission of foodborne illness to humans (Fancher et al. 2020).

**Table 1:** Key bacterial groups naturally found in poultry gut microbiota

Category	Bacterial genus/Group examples	Key roles/Impacts as natural gut residents	References
Considered beneficial residents	*Lactobacillus* spp.	Often abundant; produce lactic acid (lowers pH, inhibits some pathogens); compete with pathogens; modulate immunity.	Adhikari et al. (2020)
	*Bifidobacterium* spp.	Contribute to SCFA production; involved in immune modulation (often anti-inflammatory); common in ceca.	Idowu et al. (2025)
	*Clostridium* spp. (beneficial clusters, e.g., IV, XIVa)	Includes important butyrate producers (e.g., *C. butyricum*); butyrate is key energy source for gut cells; involved in immune regulation.	Pan and Yu (2014)
	*Bacillus* spp.	Some resident species produce antimicrobial substances; compete with pathogens; enhance gut barrier.	Idowu et al. (2025)
	*Enterococcus* spp.	Some resident species produce bacteriocins; compete with pathogens.	Yaqoob et al. (2022)
	Other beneficial residents (*Faecalibacterium*, *Ruminococcus*, etc.)	Contribute to SCFA production, fibre digestion, overall gut health.	Pan and Yu (2014)
			
Potentially undesirable/Pathogenic residents	*Salmonella enterica* (various serovars)	Can colonise gut (often asymptomatically); major cause of foodborne illness (salmonellosis) via contaminated poultry products; some serovars cause systemic disease (e.g., fowl typhoid) in poultry.	Pan and Yu (2014)
	*Campylobacter* spp. (*C. jejun*i, *C. coli*)	Common gut coloniser (often high numbers, asymptomatic); leading cause of bacterial gastroenteritis in humans; readily contaminates carcasses.	Pan and Yu (2014)
	*Escherichia coli*	Abundant gut resident; includes harmless commensals but also Avian Pathogenic *E. coli* (APEC) strains causing colibacillosis (airsacculitis, pericarditis, etc.), leading to economic losses.	Fancher et al. (2020)
	*Clostridium perfringens*	Natural gut inhabitant; specific toxin-producing strains cause necrotic enteritis (NE) and gangrenous dermatitis, especially problematic post-AGP ban; potential foodborne illness risk.	Fancher et al. (2020)
	*Listeria monocytogenes*	Can be present in gut/environment; significant food safety concern, especially for RTE poultry products (can grow at refrigeration temps).	Pan and Yu (2014)
	*Staphylococcus aureus*	Can be found on skin/in environment, sometimes gut; can cause various poultry diseases (e.g., bumblefoot, arthritis, septicemia); some strains are antibiotic-resistant.	Yaqoob et al. (2022)

This table provides examples and is not exhaustive; Roles and pathogenicity can be strain-specific

APEC = Avian pathogenic *Escherichia coli*; NE = necrotic enteritis; RTE = ready-to-eat; SCFA = short-chain fatty acid(s); spp. = species (plural)

Among the various alternatives studied, bacteriophages (or phages) have recently emerged as a potential solution to address these challenges. These viruses specifically infect and lyse bacteria, offering a targeted approach compared to broad-spectrum antibiotics (Abbas et al. 2022). Phages commonly investigated for poultry applications are tailed viruses (previously classified under the order Caudovirales, now class Caudoviricetes) that exhibit common morphotypes such as myovirus, siphovirus, and podovirus and target key pathogens such as *Salmonella*, *Campylobacter*, *Escherichia coli*, and *Clostridium perfringens* (Ackermann 2003; Zbikowska et al. 2020). A critical factor influencing their success is their host range – the spectrum of bacterial strains they can infect (Zbikowska et al. 2020). This specificity, governed by interactions between phage structures and bacterial surface receptors, can vary significantly (Jorda et al. 2023). Some phages exhibit a very narrow host range, limited to specific strains within a species, while others possess a broader host range, capable of infecting multiple strains or even species (Zbikowska et al. 2020). While this specificity is advantageous for minimising disruption to beneficial gut microbes, it necessitates careful phage selection or the use of phage cocktails (mixtures of different phages) to ensure effectiveness against the diverse bacterial populations often encountered in poultry production and to mitigate the development of phage-resistant bacteria (Zbikowska et al. 2020). Table 2 summarises recent findings on the types and host range characteristics of phages commonly investigated for key poultry-associated bacteria. The potential of bacteriophage therapy lies in its ability to selectively target specific bacterial populations (pathogens or even abundant commensals), offering a way to modulate the microbiota without the broad-spectrum disruption caused by antibiotics, potentially restoring or maintaining a favourable microbial balance (Wang et al. 2013).

**Table 2:** Summary of common phage types and host range characteristics against key poultry bacteria

Target bacterium	Common phage types/Families identified	Host range general characteristics	References
*Salmonella* spp. (various serovars, e.g., Enteritidis, Typhimurium, Infantis)	Commonly siphovirus, podovirus, and myovirus morphotypes; *Ackermannviridae* family.	Often exhibit narrow, serovar-specific host ranges, necessitating cocktails for broad coverage against diverse field strains.	Thanki et al. (2023b)
*Campylobacter* spp. (*C. jejuni*, *C. coli*)	Commonly myovirus and siphovirus morphotypes.	Generally show high strain specificity (narrow range), making cocktails essential to cover diverse strains and manage resistance.	Zbikowska et al. (2020); Olson et al. (2022)
*Escherichia coli* (esp. APEC, STEC)	Commonly myovirus, siphovirus, and podovirus morphotypes.	Exhibit highly variable host ranges, from strain-specific to broad activity against multiple pathogenic strains.	Eid et al. (2022)
*Clostridium perfringens*	Commonly siphovirus, podovirus, and myovirus morphotypes.	Often show narrow host ranges, but broad-range myovirus and siphovirus (infecting >80% of strains) exist and are preferred for cocktails.	Thanki et al. (2024)

This table summarises general trends and recent findings; specific host ranges vary greatly between individual phages; Families listed are commonly reported for phages targeting these bacteria

APEC = Avian pathogenic *E. coli*; spp. = species (plural); STEC = Shiga toxin-producing *Escherichia coli*

Bacteriophages offer several advantages over AGPs, including their specificity, which helps preserve beneficial gut microbiota. They also exhibit stability in feed and water, when properly formulated, making them potentially cost-effective and easy to produce (Wernicki et al. 2017). While much research has focused on bacteriophages’ therapeutic effects against specific pathogens under challenge conditions, their potential as growth promoters in apparently healthy chickens remains less explored. Evaluating their efficacy under typical production conditions (i.e., without deliberate pathogen challenge) is crucial for their broader adoption as AGP alternatives.

Recent studies examining phage supplementation without bacterial challenges have shown mixed results. While some research demonstrates significant improvements in growth performance (Wang et al. 2013; Sarrami et al. 2022; Shaufi et al. 2023; Mohd Shaufi et al. 2024), others report no substantial benefits (Upadhaya et al. 2021; Dlamini et al. 2023). Several factors could contribute to these inconsistent findings, including differences in phage concentration, delivery methods, target specificity, and interactions with the existing gut microbiota. Because AGPs were primarily used for growth promotion in presumed healthy commercial flocks, evaluating phage efficacy under similar non-challenge conditions is crucial for assessing their practical value as alternatives. However, most research to date has focused on pathogen challenge models. This review, therefore, addresses a critical gap by specifically analysing studies evaluating the impact of phages on poultry growth performance and health in the absence of a deliberate bacterial challenge, aiming to clarify their potential role under standard production conditions.

## PHAGES: AN OVERVIEW

To fully appreciate their potential as AGP alternatives in the specified non-challenge context, a foundational understanding of bacteriophages is essential. Phages have emerged as a promising alternative to antibiotics due to their unique properties, offering several advantages for managing bacterial infections, especially in poultry production. As summarised in Table 3, phages were first discovered over a century ago, with early applications in poultry, highlighting their potential in animal health (Ackermann 2008). Their high specificity enables them to target particular bacterial strains without disrupting beneficial gut microbiota, making them ideal for use in environments where maintaining a balanced microbiome is crucial (Koonin and Starokadomskyy 2016; Azam et al. 2021).

**Table 3:** Key characteristics and properties of phages

Characteristic	Description	Relevance	References
Discovery	Discovered by Twort (1915) and d’Herelle (1917). First used for therapy against fowl typhoid in chickens.	Early use in poultry highlights potential in animal health and as an alternative to antibiotics.	Ackermann (2008)
Categories	Two types: Obligate lytic (destroy hosts) and Temperate (can integrate into host genome).	Obligate lytic phages are preferred for therapy due to their ability to kill bacterial hosts.	Wernicki et al. (2017)
Specificity	Phages are highly specific, targeting bacteria at species or strain level.	This reduces disruption of beneficial microbiota and allows targeted treatment.	Koonin and Starokadomskyy (2016); Jaglan et al. (2022)
Diversity and isolation	Phages are diverse and most bacteria have corresponding phages, making isolation simple.	Simplifies the process of finding suitable phages for different bacterial pathogens.	Azam et al. (2021)
Self-replication	Phages replicate as long as their bacterial hosts are present.	Enables use at lower dosages compared to antibiotics, reducing costs and environmental impact.	Rahimi-Midani et al. (2021)
Adaptability	Phages can evolve with their bacterial hosts, overcoming bacterial resistance.	Ensures long-term efficacy against bacterial resistance, making them sustainable alternatives.	Koskella and Brockhurst (2014); Piel et al. (2022)
Safety	Lower risk of developing resistance compared to antibiotics; safe for use in animals.	Provides a viable option for reducing antibiotic use in agriculture and managing AMR risks.	Lyon (2017); Oechslin (2018)

AMR = antimicrobial resistance

The distinction between obligate lytic and temperate phages is critical, with a preference for obligate lytic phages in therapeutic use due to their ability to destroy bacterial hosts without integrating into the bacterial genome (Wernicki et al. 2017). This feature minimises the risk of unwanted genetic transfers that could enhance bacterial virulence, a critical consideration in designing safe and effective phage therapies.

Moreover, phages’ ability to self-replicate in the presence of their bacterial hosts allows for lower dosing requirements than antibiotics (Rahimi-Midani et al. 2021). This property, along with their adaptability to bacterial evolution, supports their long-term efficacy, even as bacteria develop resistance (Koskella and Brockhurst 2014; Piel et al. 2022). Such characteristics make phages a potentially more sustainable alternative to antibiotics in managing antimicrobial resistance (AMR).

The unique properties of phages, such as specificity, self-replication, and adaptability, position them as a viable solution for reducing antibiotic use in poultry farming. However, the variability in outcomes across studies, especially under non-challenge conditions, points to the need for further research to optimise phage applications for consistent results. To better understand this potential, it is crucial to first compare phages directly with the AGPs they aim to replace, particularly concerning their mechanisms and broader impacts on the gut environment, before focusing on the specific factors influencing supplementation success.

## PHAGE VERSUS ANTIBIOTIC GROWTH PROMOTERS (AGPs)

Having outlined the basic characteristics of phages, it is pertinent to compare their operational mechanisms and overall impact with those of the AGPs they are intended to replace. Phages and AGPs share a fundamental similarity: both aim to eliminate or control bacterial populations. However, their modes of action differ significantly, influencing their overall impact on poultry growth and health. Phages adopt a highly targeted approach, infecting specific bacterial strains, while AGPs typically exhibit broad-spectrum activity, affecting a wider range of bacterial species. This difference in specificity means that phages can selectively reduce pathogenic or non-beneficial bacteria, preserving the beneficial gut microbiota, whereas AGPs may disrupt both harmful and beneficial bacteria (Upadhaya et al. 2021; Shaufi et al. 2023). Such specificity makes phages potentially less disruptive to the gut ecosystem compared to AGPs.

Table 4 compares the mechanisms of action of AGPs and phages, highlighting the differences in their effects. For example, AGPs promote growth through a range of indirect mechanisms, such as reducing microbial competition for nutrients and enhancing nutrient absorption by altering gut morphology (Feighner and Dashkevicz 1987; Lin 2014). In contrast, phages target specific pathogenic bacteria, such as *Salmonella* or *E. coli*, directly reducing their populations without broadly affecting the rest of the gut microbiota (Lusiak-Szelachowska et al. 2017).

**Table 4:** Comparison of mechanisms of action: AGPs vs. Phages

Mechanism	AGPs	Phages	References
Bacterial targeting	Broad-spectrum; affects both harmful and beneficial bacteria.	Highly specific; targets particular bacterial strains without disrupting beneficial microbiota.	Lusiak-Szelachowska et al. (2017); Upadhaya et al. (2021)
Reduction of Gram-positive bacteria	Reduces populations linked to certain diseases.	Targets specific strains if associated with pathogenic activity.	Feighner and Dashkevicz (1987); Wernicki et al. (2017)
Reduction of gut microbial toxic by-products	Lowers toxic by-products from gut bacteria.	Decreases specific bacterial populations that produce toxins.	Feighner and Dashkevicz (1987); Marongiu et al. (2021)
Nutrient competition in the gut	Reduces overall microbial competition for nutrients.	Specifically targets nutrient-competitive pathogenic strains, preserving beneficial microbes.	Feighner and Dashkevicz (1987); Stern et al. (2012)
Enhancement of nutrient absorption	Increases absorption through thinning of the intestinal wall.	Indirectly improves nutrient absorption by reducing competition from pathogenic strains.	Feighner and Dashkevicz (1987); Shaufi et al. (2023)
Alteration of micro-biota composition	Can enrich butyrate- and lactic acid-producing bacteria.	Promotes a balanced microbiota by selectively reducing harmful strains, allowing beneficial microbes to thrive.	Robinson et al. (2019); Shaufi et al. (2023)
Impact on microbiota functionality	Alters energy and amino acid synthesis pathways without major structural changes to microbiota.	Maintains microbiota structure while eliminating specific pathogens, potentially leading to improved metabolism.	Hodak et al. (2023); Wernicki et al. (2017)

AGP = antibiotic growth promoter; spp. = species (plural)

Phages present a promising potential to address some of the mechanisms attributed to AGPs through more precise actions. For example, phages can reduce harmful bacterial populations, decrease microbial toxins, and limit nutrient competition in the gut, thereby indirectly promoting the growth of beneficial bacteria (Stern et al. 2012; Marongiu et al. 2021). Additionally, phage application can foster a more balanced gut environment by promoting the growth of beneficial butyrate-producing bacteria, similar to the effects seen with certain AGPs (Shaufi et al. 2023). These targeted actions suggest that phages could offer a more sustainable approach to improving growth performance in poultry, especially as the industry moves away from AGPs.

## GENERAL APPLICATIONS OF BACTERIOPHAGES IN POULTRY PRODUCTION

Before specifically examining the effects of phage supplementation on broiler performance in the absence of defined challenges, this section provides an overview of the diverse applications of bacteriophages in the broader context of poultry production. Understanding these general uses and their associated challenges offers valuable insights relevant to their application as growth promoters.

Phage therapy, the use of phages to treat active infections in live birds, remains a primary focus, particularly against multi-drug resistant (MDR) strains where conventional antibiotics fail. Studies demonstrate significant reductions in pathogen loads (e.g., *Salmonella*, *C. perfringens*, APEC) and associated improvements in clinical signs or mortality. However, efficacy can be variable, influenced by factors like phage selection (cocktails are often essential), dosage, timing, delivery route (e.g., feed, water, aerosol), host factors, and the dynamic emergence of bacterial resistance (Colavecchio and Goodridge 2017; Zbikowska et al. 2020; Abbas et al. 2022). Prophylactic application, administering phages pre-emptively via feed or water to reduce pathogen colonisation and shedding before significant challenges arise, is also a key strategy, particularly for food safety pathogens like *Salmonella* and *Campylobacter*. Delivering phages via feed has shown promise for effective prophylactic control, though the optimal route depends on the specific context (Wernicki et al. 2017; Mosimann et al. 2021).

Beyond live animal applications, phages are utilised as biocontrol agents post-harvest to enhance food safety. This involves applying phage preparations directly to poultry carcasses or meat products to reduce surface contamination by pathogens such as *Salmonella*, *Listeria*, and *Campylobacter* (Zbikowska et al. 2020). Several commercial products have achieved regulatory milestones, such as GRAS status in the US, facilitating their use as processing aids (Jorda et al. 2023). However, their efficacy is primarily limited to surface decontamination. Environmental biosanitation represents another critical application, where phages are used to decontaminate poultry houses, litter, and equipment, targeting persistent environmental reservoirs and potentially disrupting biofilms. Recent field studies highlight the potential of integrating phage application with conventional cleaning and disinfection (C&D) protocols, demonstrating enhanced elimination of persistent MDR *Salmonella* Infantis from farm environments (Jorda et al. 2023).

Finally, synergistic strategies are being explored, recognising that combining phages with other interventions may offer enhanced control. This includes the standard use of phage cocktails and emerging combinations with agents like bacteriocins or probiotics, although more research is needed to optimise these combinations (Zbikowska et al. 2020; Abbas et al. 2022).

These key bacteriophage applications in poultry production are summarised in Table 5. Despite the promise across these applications, significant interconnected challenges remain regarding phage stability (necessitating costly formulations like lyophilisation or encapsulation), the potential for bacterial resistance development (driving the need for complex cocktails), navigating complex and often unclear regulatory frameworks, and ensuring cost-effectiveness for widespread adoption (Jorda et al. 2023; Jiang et al. 2024). While these diverse applications primarily address specific pathogen challenges, this review will now focus specifically on the less-explored effects of phage supplementation on broiler performance and health in the absence of such defined challenges.

**Table 5:** Summary of key bacteriophage applications in poultry production

Application area	Goal/Mechanism	Key target pathogens	Examples/Delivery/Key findings	Selected recent references
Phage therapy	Treat active infections, esp. MDR strains; alternative to antibiotics.	*Salmonella* spp., *E. coli* (APEC), *C. perfringens*, *Campylobacter* spp.	Reduce pathogen load & mortality; cocktails often needed; efficacy variable, influenced by dose/timing/delivery (feed, water, aerosol) & resistance.	Mosimann et al. (2021)
Prevention/Prophylaxis	Reduce pre-harvest colonisation & shedding; prevent disease establishment.	*Salmonella* spp., *Campylobacter* spp.	Administer via feed/water before challenge; reduce pathogen load entering processing; feed delivery promising, efficacy variable.	Wagenaar et al. (2005); Mosimann et al. (2021)
Food biocontrol	Decontaminate carcasses, meat, RTE foods post-harvest; enhance food safety.	*Salmonella* spp., *Listeria monocytogenes*, *Campylobacter* spp.	Apply via spray/dip; reduce surface contamination; several commercial products with regulatory approval e.g., GRAS in US).	Dec et al. (2020); Zbikowska et al. (2020); Jorda et al. (2023)
Environmental biosanitation	Sanitise farm/hatchery/processing environments; reduce reservoirs/biofilms.	*Salmonella* spp. (incl. MDR, e.g., *S*. Infantis), *E. coli.*	Apply via spray/wash; integrate with C&D protocols; effective against persistent MDR strains/biofilms.	Zbikowska et al. (2020); Abbas et al. (2022); Jorda et al. (2023)
Synergistic use	Enhance efficacy; overcome resistance; broaden spectrum.	Various (depends on combination).	Phage cocktails standard; potential with bacteriocins, probiotics, C&D chemicals.	Zbikowska et al. (2020); Jorda et al. (2023)

APEC = Avian pathogenic *E. coli*; C&D = cleaning and disinfection; GRAS = generally recognised as safe; MDR = multidrug-resistant; RTE = ready-to-eat; spp. = species (plural)

## COMPARATIVE ANALYSIS OF PHAGE EFFICACY ON CHICKEN GROWTH PERFORMANCE, GUT MICROBIOTA, AND HEALTH

The use of bacteriophages (phages) as alternatives to antibiotics in poultry production has gained attention due to concerns about antimicrobial resistance (AMR). This analysis critically reviews studies on the effects of phages on growth performance, gut microbiota, and overall health of chickens without the presence of external bacterial challenges. These are the only five studies to explore phage supplementation in this context, making their findings particularly relevant for understanding the potential of phages in typical poultry production environments. The studies have been categorised into those showing significant improvement in growth performance (Table 6) and those without significant effects (Table 7).

**Table 6:** Summary of effective bacteriophage applications in poultry production with significant improvement in growth performance

Source of phage	Target organism(s)	No. of phages	Method of delivery	Application regimen	Effective phage concentration	Growth performance	Gut microbiota effects	Other observations	References
Chicken intestines (Serdang, Malaysia)	*Escherichia coli*	4	Freeze-dried in feed.	*Ad libitum*	0.10% at 1.00 × 10¹⁰ PFU/g	Significant reduc-tion in FCR across days 1–21, 22–35, and 1–35.	No significant improvement in *E. coli*, *Lactobacillus*, *Bifido-bacterium* and *C. perfringens*.	Significant reduction in cholesterol; No significant physiological stress (indicated by no significant difference of H : L); No significant differences in serum IgA, IgY and IgM	Shaufi et al. 2023); Mohd Shaufi et al. (2024)
ProBe-Bac (Pathway intermediates, Seoul, South Korea)	*Salmonella* sp., *Escherichia coli*	not mentioned	Freeze-dried in feed.	*Ad libitum*	0.15% at 2.04 × 10⁸ PFU/g	Significant reduction in FCR across days 25–39 and 0–39 compared to the PC (colistin).	Significant increase in *Lactobacillus;* Significant reduction in coliform.	Significant increase in propionate; Significant increase in VH : CD; Significant increase in total antibody; Significant increase in bursa of Fabricius weight.	Sarrami et al. (2022)
Not stated	*Salmonella* Gallinarum, *Salmonella* Typhimurium, *Salmonella* Enteritidis	3	Freeze-dried in feed.	*Ad libitum*	0.05% at 1.00 × 10^8^ PFU/g	Significant reduc-tion in FCR during days 1–14.	Significant increase in *Lactobacillus* compared to NC and PC (bacitracin); Significant reduction in *E. coli* and *Salmonella*.	No significant differences in meat colour, organ weight, RBC, WBC, and lymphocyte.	Wang et al. (2013)

All results are compared to NC (negative control) unless otherwise stated as compared to PC (positive control); All significant results mentioned in the table correspond to *P* < 0.05

FCR = feed conversion ratio; H : L = heterophil-to-lymphocyte ratio; IgA = immunoglobulin A; IgM = immunoglobulin M; IgY = immunoglobulin Y; PFU/g = plaque forming units per gram; RBC = red blood cell; sp. = species (singular); VH: CD = villus height: crypt depth ratio; WBC = white blood cell

**Table 7:** Summary of bacteriophage applications in poultry production with no significant improvement in growth performance

Source of phage	Target organism(s)	No. of phages	Method of delivery	Application regimen	Phage concentration	Growth performance	Gut microbiota effects	Other observations	References
Sewage water	*Salmonella* Enteritidis, *Salmonella* Typhimurium	5	Encapsu-lated in feed.	Days 1–2, 11–12, 21–22, and 29–30	0.007 5%, 0.010 0%, and 0.017 5% at 1.00 × 10⁸ PFU/g	No significant improvement in FCE.	Not available.	Significant increase in jejunum VH : CD compared to NC and PC (bacitracin).	Dlamini et al. (2023)
CJ Cheiljedang Corp. (Seoul, South Korea)	*Salmonella* Gallinarum, *Salmonella* Typhimurium, *Clostridium perfringens*	5	Freeze-dried in feed.	*Ad libitum*	0.05% and 0.10% at 1.00 × 10⁸ PFU/g for all phages except *C. perfringens* at 1.00 × 10⁶ PFU/g	No significant improvement in FCR.	No significant improvement in *Lactobacillus, E. coli, C. perfringens* and *Salmonella*.	No significant differences in meat quality and organ weight.	Upadhaya et al. (2021)

All results are compared to NC (negative control) unless otherwise stated as compared to PC (positive control); All significant results mentioned in the table correspond to *P* < 0.05

FCE = feed conversion efficiency; FCR = feed conversion ratio; PFU/g = plaque forming units per gram; VH : CD = villus height: crypt depth ratio

The comparative analysis examines key metrics such as reductions in Feed Conversion Ratio (FCR), changes in gut microbiota composition (e.g., increases in beneficial bacteria like *Lactobacillus*), and health indicators including cholesterol levels, immune responses, and gut morphology improvements [e.g., villus height-to-crypt depth ratio (VH : CD)]. This review highlights key factors influencing phage outcomes, including phage concentration, delivery methods, and target specificity, and offers insights into the conditions under which phage supplementation can be most effective in enhancing poultry health and productivity.

### Phage concentration, method of delivery, application regimen, and number of phages

The effectiveness of phage therapy in poultry production is highly influenced by the phage concentration, delivery method, application regimen, and number of phages used. Studies with higher concentrations, such as Shaufi et al. (2023) and Mohd Shaufi et al. (2024), utilised 0.10% at 1.00 × 10^10^ PFU/g, achieving significant reductions in feed conversion ratio (FCR) across multiple growth periods. However, there was no significant improvement in gut microbiota populations of *E. coli*, *Lactobacillus*, *Bifidobacterium*, or *C. perfringens*. This concentration is higher than in other studies, and it likely provided sufficient active phages to maintain pressure on gut bacterial populations over time. A high concentration, such as 1.00 × 10^10^ PFU/g, ensures that enough phages survive the digestive process and reach the gut in quantities that can effectively target and reduce specific bacterial strains.

This is particularly important in environments with dense bacterial communities, where lower phage numbers might struggle to achieve the desired impact. Additionally, the freeze-dried delivery method and *ad libitum* regimen used in these studies ensured continuous phage availability in the feed, facilitating consistent exposure for the poultry. The combination of a high concentration and a steady delivery method likely contributed to the success observed in these studies by maintaining a stable population of active phages that could continually interact with the gut microbiota, thereby improving nutrient absorption and growth efficiency.

Sarrami et al. (2022) used a slightly lower concentration (0.15% at 2.04 × 10^8^ PFU/g), but still observed significant improvements in growth metrics during later growth phases (days 25–39). The *ad libitum* delivery and freeze-dried method used in this study may have compensated for the lower concentration by providing a consistent phage presence throughout the growth period. This suggests that while higher concentrations are beneficial, consistent delivery can also be crucial to the success of phage therapy by ensuring that active phages remain continuously available to interact with gut bacteria.

Wang et al. (2013) used a similar concentration to that in Upadhaya et al. (2021), at 0.05% at 1.00 × 10^8^ PFU/g, but unlike Upadhaya et al. (2021), Wang et al. (2013) observed significant improvements in FCR during early growth periods (days 1–14). One key difference between these studies could be attributed to the bird genetics used. Upadhaya et al. (2021) used Ross 308, while Wang et al. (2013) used Arbor Acres. While bird genetics may influence responses, a detailed comparison of genetic backgrounds across these specific studies is beyond the scope of this review. Variation across genetic lines, combined with potential differences in gut microbiota and phage-host interactions, may explain the contrasting results. Further investigation into how different broiler strains respond to phage supplementation would help optimise the use of phages in poultry production.

On the other hand, Upadhaya et al. (2021), although using freeze-dried phages in feed and an *ad libitum* regimen, reported no significant improvements in FCR. The study’s concentration of 0.05% to 0.10% at 1.00 × 10⁸ PFU/g may have been too low to exert a strong antibacterial effect, similar to Dlamini et al. (2023), who also used low concentrations and observed no improvements. The combination of a lower concentration and a potentially less effective targeting approach may not have provided sufficient active phages to impact bacterial populations effectively, resulting in no significant improvement in growth performance.

Dlamini et al. (2023) employed even lower concentrations (0.007 5% to 0.0175% at 1.00 × 10^8^ PFU/g) with an intermittent dosing regimen (administered on days 1–2, 11–12, 21–22, and 29–30), which resulted in no significant improvements in Feed Conversion Efficiency (FCE). Despite the use of encapsulated phages, which theoretically should improve survival in the acidic environment of the gastrointestinal tract, the intermittent dosing regimen may have limited their effectiveness. The sporadic exposure allowed pathogenic bacteria to regrow during periods, potentially counteracting the benefits of the phages. This highlights that encapsulation alone does not guarantee efficacy; consistent and continuous exposure is necessary to sustain phage activity in the gut environment.

When examining the number of different phages used in each study, those employing multiple phages, such as Upadhaya et al. (2021) and Dlamini et al. (2023), each with 5 phages, theoretically offered a broader range of bacterial targets. However, this benefit may be negated if the overall concentration of each phage is insufficient, as seen in these studies where no significant improvements were reported. This suggests that while diversity in phage types can be beneficial for targeting various bacteria, higher concentrations and consistent exposure are more critical for achieving positive outcomes. Beyond these application parameters, the strategic selection of bacterial targets is equally fundamental to the success of phage therapy, directly shaping its impact on the gut microbiome and subsequent host responses.

### Targeted bacteria

The specific bacteria targeted by phage therapy significantly influence the outcomes observed in these studies. Effective targeting is essential because it directly affects the composition of the gut microbiota and, consequently, the growth performance of the poultry. Shaufi et al. (2023) and Mohd Shaufi et al. (2024) did not target pathogens but instead focused on non-pathogenic *Escherichia coli* strains present in the chicken gut. This approach aimed to modulate the gut microbiome rather than directly targeting harmful pathogens. By reducing the population of *E. coli*, even non-pathogenic strains, these studies potentially reduced competition for nutrients, allowing other beneficial microbes to flourish. This may have indirectly contributed to the observed improvements in growth performance and health indicators, such as reduced cholesterol levels and stable immune markers.

The choice to target *E. coli* is supported by findings in Mohd Shaufi et al. (2015), which revealed that *Escherichia-Shigella* (which includes *E. coli*) constituted a significant proportion of the chicken gut microbiota, particularly in the ileum. At day 7, *Escherichia-Shigella* accounted for 32% of the sequences, and although its levels decreased over time, it remained at 9% by day 42. The high abundance of *E. coli* early in the birds’ development suggests that it plays a dominant role in shaping the gut environment, particularly in the ileum, where nutrient absorption is critical. As such, even non-pathogenic *E. coli* can compete with beneficial bacteria for resources, potentially affecting nutrient availability and overall gut health.

This relevance is reflected in the broader trend in the reviewed studies. Among the studies analysed, two of five that specifically targeted *E. coli* (pathogenic or non-pathogenic) reported significant improvements in growth performance. These studies include Shaufi et al. (2023) and Mohd Shaufi et al. (2024), which focused on non-pathogenic *E. coli*, and Sarrami et al. (2022), which targeted both *Salmonella* spp. and pathogenic *E. coli*. This suggests that interventions focused on managing *E. coli* populations are more likely to succeed in influencing growth outcomes than those targeting less prevalent pathogens or using insufficient phage concentrations.

In contrast, Sarrami et al. (2022) and Wang et al. (2013) targeted more classically pathogenic strains such as *Salmonella* spp., including *Salmonella* Gallinarum, *Salmonella* Typhimurium, and *Salmonella* Enteritidis. These pathogens are particularly harmful in poultry due to their impact on both animal health and food safety. The targeted elimination of these pathogens improved gut health markers, including increased *Lactobacillus* populations, reduced coliform counts, and better villus height-to-crypt depth ratios (VH : CD). These changes likely enhanced nutrient absorption, contributing to the observed reductions in FCR.

Similarly, Dlamini et al. (2023) and Upadhaya et al. (2021) targeted a combination of pathogenic *Salmonella* spp. and *Clostridium perfringens*, both of which are known to cause significant health challenges in poultry. However, these studies did not achieve significant improvements in growth performance. The lack of effectiveness may be attributed to lower concentrations and intermittent dosing regimens, which may not have been sufficient to achieve sustained reductions in these pathogenic populations. Despite targeting organisms known to have a negative impact on poultry health, inconsistent phage delivery may have allowed these pathogens to recover between doses, limiting the overall benefits of the therapy. The variability in outcomes, even when targeting known pathogens, suggests that efficacy is not solely dependent on the phage-bacterium interaction. Indeed, several overarching limitations in the design and reporting of comparative phage studies can confound results and make definitive conclusions difficult.

### Limitations in comparative phage studies

The mixed findings reported in the preceding analysis of the five key studies (Tables 6 and 7) can be attributed to several methodological and biological variables, which pose significant limitations for comparing phage efficacy across different experimental setups and for understanding the precise factors driving success or failure.

One significant limitation in many phage studies is the lack of transparency regarding stabilising additives in phage formulations. However, some recent studies have detailed their formulation methods. For example, Shaufi et al. (2023) and Mohd Shaufi et al. (2024) used a specific combination of additives in their freeze-dried phage preparation, including skim milk as a temperature protectant, maltodextrin as a cryoprotectant, and calcium carbonate (CaCO_3_) as an antacid, mixed at a ratio of 2 : 2 : 1 : 5, respectively. These additives were crucial for maintaining phage viability at ~10^10^ PFU/g throughout the study period.

The importance of protective measures is highlighted by research showing that unencapsulated phages are highly sensitive to acid, with complete inactivation occurring at pH 2.5 and below – conditions typically found in the chicken’s gizzard and proventriculus (Richards and Malik 2021). Studies have demonstrated that appropriate stabilisation methods can help maintain phage viability. The addition of CaCO_3_ to alginate formulations has been shown to significantly improve the survival of encapsulated bacteriophages in acidic conditions (Tabare et al. 2023). Various protective agents such as trehalose, sucrose, maltodextrin, and skim milk have been used as desiccant protectants for dried preparations of microencapsulated bacteriophages (Ma et al. 2012; Sliwka et al. 2019).

Variation in growth performance across studies could be attributed to differences in phage protection methods. Without proper stabilisation, phages may be denatured by the acidic environment of the gastrointestinal tract, thereby reducing their efficacy. The optimal pH range for phage activity is between pH 7 and 10, with most phages showing susceptibility below pH 4 (Liu et al. 2023; Shaufi and Sieo 2023). Therefore, studies that don’t specify their stabilisation methods or use inadequate protection may underestimate the potential benefits of phage supplementation.

Variations in feed composition and housing conditions can significantly influence gut microbiota composition and, consequently, phage-host interactions. Housing environments have been shown to explain up to 28% of caecal microbiota variation in broilers, which is substantially more than the impact of dietary interventions (Hubert et al. 2019). Studies have demonstrated that different housing systems, such as cage-free versus caged environments, can generate significant differences in gut microbiota diversity (Hubert et al. 2019). Many studies fail to report detailed environmental and operational conditions, making it difficult to determine whether variations in results stem from the phage treatment itself or from external environmental factors.

Genetic differences among poultry breeds can significantly influence gut microbiota composition, immune responses, and growth outcomes, thereby affecting the efficacy of phage treatments. For instance, Pandit et al. (2018) observed distinct variations in the caecal microbiota between indigenous Indian chicken breeds (Aseel and Kadaknath) and commercial broiler lines (Cobb400 and Ross 308), suggesting that breed-specific factors shape gut microbial communities. Additionally, Chuwatthanakhajorn et al. (2023) reported differential expression of immune-related genes following infectious bronchitis virus vaccination in Taiwan Country and White Leghorn chicken breeds, indicating breed-specific immune responses. Furthermore, Zou et al. (2020) identified genetic variants associated with immunity in broilers, highlighting that selective breeding has altered immune responses over time. These findings imply that genetic variations can affect the outcomes of phage applications, and a lack of in-depth exploration into these factors may limit the generalisability of findings across different poultry breeds.

## IMMUNE MODULATORY EFFECTS OF BACTERIOPHAGES

Beyond direct effects on growth performance and gut microbiota composition, bacteriophages also exert significant immunomodulatory effects, representing another key aspect of their impact on poultry health indicators. Recent research reveals that bacteriophages function as sophisticated modulators of the avian immune system, with capabilities that extend beyond their direct antibacterial activities. In broiler chickens, bacteriophages show significant immunomodulatory effects, particularly in regulating inflammatory responses – a crucial aspect for maintaining optimal health and growth performance in poultry production.

Advanced molecular studies have discovered that when broiler chickens infected with *Salmonella enterica* receive bacteriophage cocktail treatment, the avian immune system initially recognises bacteriophages as viral entities. However, the cGAS-STING pathway, a fundamental component of antiviral responses, is selectively inhibited at the phosphorylation of the IRF3 transcription factor. This specific interruption occurs because avian RNA polymerase III cannot effectively recognise phage DNA to produce dsRNA molecules required to activate protein complexes required for IRF3 phosphorylation (Podlacha et al. 2024). This selective pathway inhibition allows bacteriophages to eliminate target bacteria while preventing excessive inflammatory responses typically associated with infection or antibiotic treatments. Thus, bacteriophages function as precision antimicrobials that maintain gut homeostasis, a critical factor in promoting growth performance and intestinal health in poultry production.

Studies on gene expression demonstrate that bacteriophage administration significantly alters cytokine profiles in broiler chickens. Research indicates that bacteriophage supplementation enhances expression of anti-inflammatory cytokines, particularly Interleukin-10 (IL-10), in intestinal tissues (Sarrami et al. 2022). This immunological shift provides substantial benefits because IL-10 functions as a key regulator that limits excessive inflammatory processes by suppressing pro-inflammatory cytokines such as IL-12, IFN-γ, TNF-α, IL-1β, IL-2, and IL-6 (Steen et al. 2020). The resulting anti-inflammatory intestinal environment protects epithelial tissues from inflammation-induced damage, thereby enhancing gut barrier function and nutrient absorption.

Studies examining broilers under bacterial challenge conditions reveal that bacteriophage cocktails effectively modulate inflammatory responses. Researchers have documented elevated levels of anti-inflammatory mediators (IL-10 and IL-4) in birds receiving bacteriophage therapy against *Salmonella* Typhimurium (Grabowski et al. 2022). While bacteriophage treatment regulates inflammation, it maintains normal lymphocyte subpopulation numbers and activity, indicating bacteriophages can selectively control harmful inflammatory processes without compromising protective immunity, an equilibrium essential for optimal growth performance and disease resistance in poultry.

The immune-modulating capabilities of bacteriophages extend beyond cytokine regulation. Bacteriophage supplementation in broiler diets enhances humoral immunity, as demonstrated by increased serum concentrations of total antibodies, immunoglobulin M (IgM), and immunoglobulin G (IgG) compared to control groups (Sarrami et al. 2022). This enhancement of systemic antibody production occurs without triggering detrimental inflammatory cascades, suggesting bacteriophages promote protective immunity while preventing inflammatory damage.

Unlike conventional antibiotics, which frequently disrupt beneficial gut microbiota without directly influencing inflammatory mechanisms, bacteriophages preserve cytokine balance at levels comparable to those of healthy, uninfected birds. This homeostatic effect results from the gradual elimination of targeted bacteria, preventing the sudden release of large quantities of bacterial toxins typically associated with rapid antibiotic-induced bacterial lysis (Sarrami et al. 2022). This distinctive combination of targeted antimicrobial activity with sophisticated immunomodulatory capabilities positions bacteriophages as particularly valuable alternatives to antibiotic growth promoters in modern poultry production systems, aiming to reduce antimicrobial use while maintaining optimal flock health and production efficiency.

## STRATEGY FOR OPTIMISING PHAGE APPLICATION TO ENHANCE GROWTH PERFORMANCE AND HEALTH

Given the variable outcomes observed in non-challenge studies and the multifaceted nature of phage-host interactions, including the immunomodulatory effects previously discussed, optimising application strategies is paramount for achieving consistent and predictable success in enhancing growth performance and health. Key factors include phage concentration, delivery methods, dosing regimens, formulation, and the specific bacterial strains targeted (Table 8).

**Table 8:** Summary of key factors influencing phage application in poultry production

Strategy	Details	Reference
Phage concentration	High concentrations (e.g., 0.10% at 1.00 × 10¹⁰ PFU/g) improve FCR, but may increase costs in commercial production.	Torres-Acosta et al. (2019); Shaufi et al. (2023); Mohd Shaufi et al. (2024)
Delivery method	Freeze-dried phages mixed in feed ensure viability through digestion and consistent delivery.	Kuzminska-Bajor et al. (2023)
Dosing regimens	Continuous/*ad libitum* dosing yields better results than intermittent dosing for gut health.	Sarrami et al. (2022); Dlamini et al. (2023)
Formulation	Additives like calcium carbonate, skim milk, and maltodextrin preserve phage viability in the acidic gastrointestinal tract.	Vila et al. (2024)
Targeted bacteria	Targeting *E. coli* and *Salmonella* spp. improves gut health and nutrient absorption.	Sarrami et al. (2022); Shaufi et al. (2023); Mohd Shaufi et al. (2024)

FCR = feed conversion ratio; PFU/g = plaque forming units per gram; spp. = species (plural)

### Optimising phage concentration

The primary strategy for enhancing phage efficacy involves optimising phage concentration. Studies have shown that higher phage concentrations, such as 0.10% at 1.00 × 10^10^ PFU/g, significantly improve Feed Conversion Ratio (FCR) in poultry. Shaufi et al. (2023) and Mohd Shaufi et al. (2024) demonstrated that this concentration leads to substantial reductions in FCR, indicating enhanced feed efficiency. However, while these high-titre concentrations deliver significant performance improvements, scaling them for commercial poultry production can be costly (Torres-Acosta et al. 2019).

### Enhancing delivery methods

Strategically refining delivery methods is crucial for maximising phage performance. Freeze-dried phages mixed into feed have proven both effective and economical, ensuring that phages remain viable throughout the digestive process and are delivered in sufficient quantities to positively impact gut bacteria. Studies have demonstrated that phages can survive better when mixed with feed, largely due to the protective effect of feed components. For instance, research showed that when phages were tested in Simulated Gastric Fluid (SGF) containing 30% chicken feed, they exhibited better survival than in control SGF, which represented natural stomach acidity (Kuzminska-Bajor et al. 2023). Additionally, phage titres have been found to remain stable within the feed matrix across different feeding phases (starter, grower, and finisher), with no loss in titres, highlighting the effectiveness of phage integration into feed for consistent delivery throughout the poultry growth cycle (Thanki et al. 2023a). However, it is important to ensure that phages are added after the feed manufacturing process, as the heat and high pressure used during production could affect phage viability. Therefore, commercially viable options include mixing or spraying phages onto the final feed products.

### Implementing optimal dosing regimens

Continuous or *ad libitum* dosing regimens are more effective than intermittent dosing. Maintaining consistent phage levels in the gut allows for continuous interaction with gut bacteria, promoting better gut health and improved growth outcomes. Sarrami et al. (2022) showed that continuous dosing ensures more favourable results by keeping pathogenic bacteria in check, whereas intermittent dosing can allow harmful bacteria to repopulate between treatments, limiting the effectiveness of phage therapy. Dlamini et al. (2023) similarly observed that inconsistent dosing led to suboptimal outcomes, reinforcing the need for a consistent phage presence in the gut.

### Effective formulations for phage stability

The method of delivering bacteriophages is essential for maintaining their effectiveness, especially in preserving their viability during storage and use. Additives such as calcium carbonate, skim milk, and maltodextrin, commonly used in freeze-dried formulations, help protect phages from degradation in acidic environments, such as the poultry gastrointestinal tract (Vila et al. 2024). These stabilisers ensure that sufficient active phages reach their targets while providing a more cost-effective solution than more complex encapsulation techniques. This approach optimises phage delivery and efficacy in managing poultry health.

### Refining bacterial targeting strategies

Targeting the right bacterial strains is key to optimising phage therapy in poultry. Studies have demonstrated that targeting both pathogenic and non-pathogenic bacteria, such as *Escherichia coli,* can influence gut health and performance. For instance, focusing on non-pathogenic *E. coli* strains, as seen in research by Shaufi and Sieo (2023) and Mohd Shaufi et al. (2024), helped reduce competition for nutrients, indirectly improving growth performance. In contrast, other studies, such as those by Sarrami et al. (2022) and Wang et al. (2013), targeted pathogenic *Salmonella* spp., resulting in enhanced gut health and nutrient absorption, indicated by better gut microbiota balance and increased villus height-to-crypt depth ratios. Despite targeting harmful pathogens such as *Salmonella* and *Clostridium perfringens*, some studies, including Dlamini et al. (2023), did not show significant growth improvements, likely due to lower phage concentrations and inconsistent dosing regimens.

## KEY RISK AND MITIGATION STRATEGIES FOR PHAGE APPLICATION

While these strategies aim to optimise the benefits of phage application, it is equally crucial to proactively consider and address the potential risks associated with their widespread use in poultry production. A primary concern when using bacteriophages (phages) as standalone growth promoters in livestock is the potential for target bacteria to develop resistance, which could limit long-term effectiveness (Jiang et al. 2024). Bacteria can evolve resistance through various mechanisms, such as altering surface receptors to block phage attachment (Jiang et al. 2024). While phage specificity is advantageous over broad-spectrum antibiotics by minimising disruption to beneficial gut microbiota (Sahoo and Meshram 2024), there’s still a risk of unintended impacts on commensal populations or overall microbiome stability (Huon et al. 2020). The principal mitigation strategy involves using phage cocktails – mixtures of distinct phages targeting the same bacteria via distinct mechanisms – which significantly reduce the probability of simultaneous resistance emerging and broaden the effective host range to cover diverse bacterial strains (Yang et al. 2020; Jiang et al. 2024).

Safety considerations include the risk of horizontal gene transfer (HGT) and endotoxin release. Phages, especially temperate ones, can transfer undesirable genes, such as antibiotic resistance genes (ARGs) or virulence factors, between bacteria in the gut (Sun et al. 2019; Jiang et al. 2024). This risk is significantly reduced by selecting and using only obligately lytic phages, as confirmed by genomic screening for the absence of genes associated with lysogeny or unwanted traits (Zalewska-Piatek 2023; Jiang et al. 2024). Additionally, the lysis of Gram-negative bacteria inherently releases lipopolysaccharides (LPS or endotoxins), which can trigger harmful inflammatory responses (Ling et al. 2022). This necessitates rigorous purification of phage preparations during manufacturing, using methods like affinity chromatography or ion exchange, to remove endotoxin contaminants and ensure product safety (Hietala et al. 2019).

Significant practical challenges also hinder the widespread adoption of phages as feed additives. Phages are sensitive to environmental stresses, such as heat during feed pelleting and the acidic conditions of the stomach, which can drastically reduce their viability (Ma et al. 2008; Richards and Malik 2021). Encapsulation technologies, such as using pH-responsive polymers or alginate-based matrices, are crucial for protecting phages and ensuring targeted delivery to the intestines (Richards and Malik 2021; Yang et al. 2023b). Furthermore, achieving cost-effectiveness compared to other alternatives, scaling up production under Good Manufacturing Practice (GMP) standards with consistent quality control, and navigating complex, often unclear regulatory pathways in regions like the US (Food and Drug Administration (FDA)) and EU (European Food Safety Authority (EFSA)) remain major hurdles requiring ongoing development and legislative efforts (Bretaudeau et al. 2020; Yang et al. 2023a).

## CONCLUSION

Bacteriophages offer a compelling alternative to antibiotic growth promoters (AGPs) for broiler chickens, especially in non-challenge production environments, where their success hinges on specific, controllable factors. Evidence indicates that achieving significant improvements in growth performance and gut health, such as Feed Conversion Ratio (FCR), requires high phage concentrations (e.g., ≥10^8^ PFU/g), continuous delivery regimens, and protective formulations. Strategic bacterial target selection—whether reducing pathogens like *Salmonella* or modulating abundant commensals such as *E. coli* to potentially enhance nutrient availability – also profoundly impacts outcomes. Beyond direct lysis, phages exhibit distinct immunomodulatory capabilities, promoting beneficial anti-inflammatory responses and enhancing systemic immunity, unlike antibiotics. However, realising this potential necessitates navigating challenges such as bacterial resistance, ensuring product safety from endotoxins and risks of horizontal gene transfer, and overcoming practical hurdles in formulation, cost, and regulation. Therefore, future research must prioritise establishing standardised protocols, detailed reporting, and deeper investigation into host genetic and environmental interactions. With continued focused research and strategic optimisation, bacteriophages are poised to become a vital, sophisticated tool in sustainable poultry production, significantly contributing to broiler health and performance in the post-AGP era.
